# Benfotiamine upregulates antioxidative system in activated BV-2 microglia cells

**DOI:** 10.3389/fncel.2015.00351

**Published:** 2015-09-04

**Authors:** Iva Bozic, Danijela Savic, Ivana Stevanovic, Sanja Pekovic, Nadezda Nedeljkovic, Irena Lavrnja

**Affiliations:** ^1^Institute for Biological Research “Siniša Stanković,” University of BelgradeBelgrade, Serbia; ^2^Institute for Medical Research, Military Medical AcademyBelgrade, Serbia; ^3^Faculty of Biology, Institute for Physiology and Biochemistry, University of BelgradeBelgrade, Serbia

**Keywords:** benfotiamine, microglia, LPS, oxidative stress, catalase, glutathione

## Abstract

Chronic microglial activation and resulting sustained neuroinflammatory reaction are generally associated with neurodegeneration. Activated microglia acquires proinflammatory cellular profile that generates oxidative burst. Their persistent activation exacerbates inflammation, which damages healthy neurons via cytotoxic mediators, such as superoxide radical anion and nitric oxide. In our recent study, we have shown that benfotiamine (*S*-benzoylthiamine O-monophosphate) possesses anti-inflammatory effects. Here, the effects of benfotiamine on the pro-oxidative component of activity of LPS-stimulated BV-2 cells were investigated. The activation of microglia was accompanied by upregulation of intracellular antioxidative defense, which was further promoted in the presence of benfotiamine. Namely, activated microglia exposed to non-cytotoxic doses of benfotiamine showed increased levels and activities of hydrogen peroxide- and superoxide-removing enzymes—catalase and glutathione system, and superoxide dismutase. In addition, benfotiamine showed the capacity to directly scavenge superoxide radical anion. As a consequence, benfotiamine suppressed the activation of microglia and provoked a decrease in NO and ^·^O^−^_2_ production and lipid peroxidation. In conclusion, benfotiamine might silence pro-oxidative activity of microglia to alleviate/prevent oxidative damage of neighboring CNS cells.

## Introduction

Neurons are very susceptible to oxidative stress, as a result of great metabolic rate, large oxygen consumption, relatively weak antioxidative defense, low regenerative capacity, and specific cellular geometry (Andersen, 2004; Barnham et al., 2004). Current knowledge of neurological disorders, from ischemia and brain injury to psychiatric disorders and neurodegenerative diseases, indicates that pathological mechanisms involve acute or chronic activation of microglia and resulting overproduction of reactive oxygen (ROS) and nitrogen species (RNS) by the cells (Uttara et al., 2009; Rojo et al., 2014). These immune cells of the brain are on the constant patrol for invading pathogens and metabolic, ischemic or traumatic brain damage (Aguzzi et al., 2013; Gertig and Hanisch, 2014). Upon receiving the pathogen- or danger-associated signals, microglia promptly activate, acquire amoeboid morphology, migrate toward the site of damage and release an array of noxious, proinflammatory mediators, such as cytokines, superoxide radical anions (superoxide; ^·^O^−^_2_), hydrogen peroxide (H_2_O_2_), and nitric oxide (NO) (Block et al., 2007). Although microglial activation aims for removal of potential threats from the damaged tissue, it also harms surrounding healthy tissue via ROS production and lipid peroxidation, which affect structural proteins and enzymes, RNA and DNA, the integrity of membranes, and mitochondrial membrane potential (Li et al., 2013; González et al., 2014). Apart from this, as proliferation and pro-oxidative activity of microglia appear to be propagated by ROS (Jekabsone et al., 2006; Mander et al., 2006), these species potentiate microglial activity in feed-forward manner and exacerbate inflammation (Min et al., 2004; Barger et al., 2007; Rojo et al., 2014). Modulation and suppression of microglia activation has been shown to alleviate symptoms in various neurological conditions that have been related to hyper-reactive microglia, such as brain injury (Roth et al., 2014), multiple sclerosis (Giunti et al., 2014), Alzheimer's disease (Solito and Sastre, 2012), Parkinson's disease (Van der Perren et al., 2015), and amyotrophic lateral sclerosis (Frakes et al., 2014). In order to bring under control oxidative burst exerted by microglia on its surroundings in the CNS, two potential strategies might be applied: (i) direct scavenging/detoxification of ROS; (ii) up/down-regulation of endogenous systems for removal/formation of reactive oxygen species (Kumar et al., 2014; Miljkovic et al., 2015). It is noteworthy that both strategies already offered some promising results. For instance, intracranial application of glutathione (GSH) modulates microglia activity and improves clinical outcomes of brain injury (Roth et al., 2014). Similarly, a novel drug for treatment of multiple sclerosis—dimethyl fumarate, affects ROS and RNS production by microglia via upregulation of intracellular antioxidative system (Wilms et al., 2010; Lin et al., 2011).

Benfotiamine (*S*-benzoylthiamine O-monophosphate) is an S-acyl derivative of thiamine (vitamin B_1_) (Fujiwara, 1954) and is easily absorbed with good bioavailability and safety profile (Bitsch et al., 1991; Balakumar et al., 2010). Favorable effects of benfotiamine have been already documented in the treatment of diabetic and alcoholic neuropathies (Hammes et al., 2003; Balakumar et al., 2010; Manzardo et al., 2013). Mechanisms of benfotiamine action include antioxidative and anti-inflammatory effects as documented under both, *in vitro* and *in vivo* settings (Ceylan-Isik et al., 2006; Wu and Ren, 2006; Balakumar et al., 2008; Schmid et al., 2008; Schupp et al., 2008; Verma et al., 2010; Harisa, 2013), as well as in patients suffering from diabetes type II (Stirban et al., 2006). We have recently shown that benfotiamine attenuates microglial activation by altering cell morphology and suppressing the production of proinflammatory mediators. These effects were mediated via nuclear factor kappa-B (NF-κB) and MAPK signaling, on which benfotiamine exerted direct effects (Bozic et al., 2015).

Therefore, benfotiamine represents an off-the-shelf agent, whose potential applications might expand to other conditions that are related to microglia “outrage”. Here we examined the effects of benfotiamine on pro-oxidative activity of activated BV-2 microglia cells. The focus was on the main components of the endogenous antioxidative system: catalase (CAT), cytoplasmic and mitochondrial superoxide dismutases (SOD1 and SOD2, respectively), along with total glutathione and enzymes involved in its metabolism, GSH peroxidase (GPx) and reductase (GR).

## Materials and methods

### Cell culture and treatment

BV-2 cell line was derived from primary microglial cells of C57BL/6 newborn mice infected with v-raf/v-myc retrovirus (Blasi et al., 1990). These cells express functional NADPH oxidase (Henn et al., 2009). They were a kind gift from Dr. Alba Minelli from the University of Perugia, Italy. BV-2 microglia was cultured in RPMI 1640 medium (GE Healthcare Life Sciences, Freiburg, Germany) containing 10% heat-inactivated fetal bovine serum (FBS, PAA Laboratories GmbH, Pasching, Austria) and 1% penicillin/streptomycin (Invitrogen Co, Carlsbad, CA, USA) at 37°C in a humidified incubator with 5% CO_2_. Upon confluence, cells were collected with 0.1% trypsin-EDTA (PAA Laboratories GmbH, Pasching, Austria), centrifuged (500 × g for 5 min) and seeded in culture dishes, depending on the experiment. Cell were exposed to benfotiamine (50, 100, 250 μM; Sigma-Aldrich Labware, Munich, Germany) 30 min prior to stimulation with 1 μg/ml LPS (Escherichia coli serotype 026:B6; Sigma-Aldrich Labware, Munich, Germany) for 24 h or as indicated. Treatment with inhibitor of inducible NO synthase (iNOS), Nω-Nitro-L-arginine methyl ester hydrochloride (L-NAME) was performed 1 h before BV-2 cells were incubated with LPS for 24 h.

### Cell viability assay

For MTT test, cells were grown in 96-well plates (1 × 10^4^ cells/well), exposed to benfotiamine and LPS for 24 h, as previously described (Bozic et al., 2015). The test based on a reduction of 3-(4,5-dimethylthiazol-2-yl)-2,5-diphenyltetrazolium bromide (MTT) to formazan is an indicator of total mitochondrial status of viable cells. Cells were incubated with MTT solution (5 mg/ml) for 30 min at 37°C. Purple crystals of formazan were dissolved in DMSO and absorbance was measured at 492 nm with a microplate reader (LKB 5060-006, Vienna, Austria). The results are expressed as % of mean optical density (OD 492 nm) relative to control ± SEM, from three independent determinations performed in triplicate.

### Flow cytometric analysis

Cells were seeded in 6-well plates (3 × 10^5^ cells/well) and treated as described. They were detached with 0.1% trypsin-EDTA, centrifuged at 750 × g for 3 min and washed twice with phosphate buffered saline (PBS). Subsequently, the cells were stained with FITC-conjugated anti-mouse CD40 antibody or isotype control (1:200, BD Pharmingen) for 1 h at 4°C. BV-2 microglia was spun down, rinsed twice with PBS and analyzed with CyFlow® Space Partec (Partec GmbH, Munster, Germany) using PartecFloMax®software (Partec GmbH, Munster, Germany). Minimum of 10,000 cells were analyzed and results are presented as % (dot plots) and number (graph) of cells expressing CD40.

For determination of apoptotic and necrotic cell death, cells were incubated with annexin V FITC and propidium iodide (Molecular Probes, Invitrogen, Carlsbad, CA) for 15 min in the dark at room temperature (RT). Annexin V binds to phosphatidylserine that is exposed on the outer leaflet of plasma membrane of apoptotic (and at least in some cases necrotic) cells. Propidium iodide is a DNA intercalating agent that can enter only necrotic cells. Thus, cells positive only for annexin V were identified as apoptotic, whereas cells that were positive for propidium iodide and annexin V were recognized as necrotic. The reaction was stopped by centrifuging cells and resuspending them in Annexin Binding Buffer. Analysis was performed at CyFlow® Space Partec using PartecFloMax® software.

### Radical generating systems

Radical-generating systems were prepared as described previously (Miljkovic et al., 2015). The ability of benfotiamine to scavenge hydroxyl radical (^·^OH) was tested using the Fenton system (Fe^2+^ + H_2_O_2_ → Fe^3+^ + OH^−^ + ^·^OH). The Fenton reaction was performed in PBS (pH = 7.4) by combining 1 mM of H_2_O_2_ (Renal, Budapest, Hungary) and 0.2 mM of FeSO_4_(Merck, Darmstadt, Germany). Spin trap DEPMPO (5-diethoxyphosphoryl-5-methyl-1-pyrroline-*N*-oxide; Enzo Life Sciences International, Plymouth Meeting, PA, USA; 10 mM final concentration) was added prior to H_2_O_2_. The time period between the initiation of reaction and EPR measurements was 2 min. Benfotiamine was supplemented before the initiation of reaction at the final concentration of 1 mM.

Superoxide was generated using ^·^O^−^_2_ thermal source SOTS-1 (Cayman Chemicals, Ann Arbor, MI, USA). Immediately prior to the start of any experiment the SOTS-1 was dissolved in DMSO, and was further supplemented to PBS solution containing DEPMPO (10 mM) and DTPA (1 mM; chelating agent, which is added in order to suppress the redox activity of transition metals impurities in ^·^OH-generating Haber-Weiss reaction), to a final concentration of 0.2 mM. This system was incubated for 5 min at 37°C. The time period between the end of incubation and EPR measurements was 2 min.

### EPR spectroscopy

EPR measurements were performed on X-band (9.57 GHz) Varian E104-A EPR spectrometer, using EW software (Scientific Software, Bloomington, IL, US). Settings were: 10 mW, microwave power; 2 G, modulation amplitude; 3410 G, field center; 200 G, scan range; 4 min, scanning time; 100 kHz, modulation frequency; 32 ms, time constant. Samples were drawn into gas-permeable Teflon tubes (internal diameter 0.6 mm; wall thickness 0.025 mm; Zeus industries, Raritan, NJ, USA) to maintain constant oxygen level in the sample, Teflon tubes were placed in quartz capillaries. DEPMPO reacts with ^·^OH and ^·^O^−^_2_ producing DEPMPO/OH and DEPMPO/OOH adducts, respectively. Signal intensities were determined using spectral simulations, which were conducted using WINEPR SimFonia computer program (Bruker Analytische Messtechnik GmbH, Darmstadt, Germany). Simulation parameters were: (i) DEPMPO/OH adduct: aN = 13.64 G; aH = 12.78 G; aP = 46.7 G; (ii) DEPMPO/OOH adduct: isomer I (50%): aN = 13.4 G, aH_β_ = 11.9 G, aH_γ_ (1H) = 0.8 G, aH_γ_ (6H) = 0.43 G, aP = 52.5 G; isomer II (50%): aN = 13.2 G, aH_β_ = 10.3 G, aH_γ_ (1H) = 0.9 G, aH_γ_ (6H) = 0.43 G, aP = 48.5 G. Antioxidative activity (AA) was calculated using the following formula: (I_0_ - I_*x*_)/I_0_, where I_0_ and I_x_ are the intensities of EPR spectra obtained in control and samples with benfotiamine. Maximal AA value is 1. *EC*_50_-value (mM) is the effective concentration at benfotiamine provoked 50% decrease in DEPMPO/OOH yield, obtained by interpolation from linear regression analysis. Each experiment was performed in triplicate. In addition, the rate constant for benfotiamine reaction with superoxide was calculated using previously described method and rate constant for the reaction between DEPMPO and superoxide (approximately 4 M^−1^ s^−1^).

### Quantitative real-time PCR

BV-2 microglia was plated (6-well plates, 3 × 10^5^ cells/well) and incubated with benfotiamine and LPS. Four hours after the onset of LPS stimulation, cells were collected for total RNA isolation in TRIzol reagent (Invitrogen, Carlsbad, CA, USA). The RNA content was quantified spectrophotometrically and the purity was evaluated by running RNA samples on agarose gels. Reverse transcription was conducted with 1 μg of RNA, with High Capacity cDNA Reverse Transcription Kit (Applied Biosystems, Foster City, CA, USA). ABI Prism 7000 Sequence Detection System (Applied Biosystems, Foster City, CA, USA) was used for real-time PCR analysis, with SYBR Green PCR Master Mix (Applied Biosystems, Foster City, CA, USA) and specific primers for CAT, SOD2 and GPx (sequences and annealing temperatures given in Table 1, Invitrogen, Carlsbad, CA, USA). Relative expression of target genes was evaluated using the 2^−ΔΔ*CT*^method, with β-actin as internal control.

**Table 1:** **List of primers used for Real-time PCR**.

**Target gene**	**Forward primer**	**Reverse primer**	**Size (*bp*)**	**Annealing T (°C)**
CAT	AGCGACCAGATGAAGCAGTG	TCCGCTCTCTGTCAAAGTGTG	181	64
MnSOD	CAGACCTGCCTTACGACTATGG	CTCGGTGGCGTTGAGATTGTT	113	64
GPx	AGTCCACCGTGTATGCCTTCT	GAGACGCGACATTCTCAATGA	105	64
Actin	GGGCTATGCTCTCCCTCAC	GATGTCACGCACGATTTCC	136	63

### Western blot

Western blot analysis was performed as previously described (Bozic et al., 2015). Briefly, after treatment cells were lysed, samples were centrifuged and protein content was determined. Equal amounts of total cellular proteins were loaded per lane of 10% (for analysis of SOD2 and GPx) and 7.5% (for analysis of CAT and GR) polyacrylamide gels. Proteins were resolved at constant voltage (100–120 V) and transferred to polyvinylidene fluoride (PVDF) support membrane (Roche, Penzberg, Germany). Support membranes were blocked with the blocking solution (5% BSA dissolved in TBST (20 mM Tris, pH 7.6, 136 mM NaCl, 0.1% Tween 20) for 1 h at RT to eliminate unspecific binding. Membranes were incubated overnight with primary antibodies (Table 2), washed 3 times for 10 min with TBST and incubated with secondary antibodies for 1 h at RT. Chemiluminescence was used to visualize antibody binding. Protein bands were analyzed using ImageQuant 5.2 software, by determining optical density of the band and normalizing it to the optical density of β-actin from the same lane. Results are expressed as mean relative target protein/β-actin abundance ± SEM, from three separate determinations.

**Table 2:** **List of primary antibodies used for flow cytometry (FC) and western blot (WB)**.

**Antigen**	**Source**	**Dilution**	**Company**
CD40	mouse	1:200 (FC)	BD Pharmingen
CAT	Rabbit	1:5000 (WB)	Abcam
GPx	Rabbit	1:5000 (WB)	Abcam
MnSOD	Rabbit	1:5000 (WB)	Abcam
GR	Rabbit	1:5000 (WB)	Abcam

### Determination of parameters involved in cell oxidative state

BV-2 cells were plated (6-well plates, 3 × 10^5^ cells/well) and stimulated for 24 h as described. Cells were rinsed with ice cold PBS and collected with a cell scraper. Cells were lysed by sonication and centrifuged at 15,000 × g for 5 min, at 4°C. Supernatants were collected and used for determination of ^·^O^−^_2_, malondialdehyde (MDA) and total glutathione (reduced + oxidized) and activity of enzymes involved in antioxidative defense, SOD2, CAT, GPx, and GR. The protein content was determined by the method of Lowry using bovine serum albumin as standard (Lowry et al., 1951).

### Measurement of NO production

BV-2 microglia was seeded in 24-well plates (5 × 10^4^ cells/well), and treated as described. Culture medium was collected, deproteinized and concentration of NO was evaluated by measuring nitrite and nitrate concentrations. Griess method was used to determine the nitrite content. Griess reagent was made of 1.5% sulfanilamide (Sigma-Aldrich, Munich, Germany) in 1 M HCl and 0.15% N-(1-naphthyl) ethylendiamine dihydrochloride (Fluka, Buchs, Switzerland) in distilled water. Nitrates were first transformed into nitrites by cadmium reduction (Navarro-Gonzálvez et al., 1998). Concentration of nitrites released in the medium was determined from the standard curve generated with known concentrations of sodium nitrite (Mallinckrodt Chemical Works—St. Louis, MO, USA). Results are expressed as mean concentration of nitrites (μM) ± SEM, from three separate determinations.

#### Superoxide anion radical

Concentration of ^·^O^−^_2_ was evaluated with the method based on the reduction of nitroblue-tetrazolium—NBT (Sigma-Aldrich—Sr. Louis, USA) to monoformazan by ^·^O^−^_2_ in the alkaline nitrogen saturated medium. The product of this reaction is yellow and was measured spectrophotometrically at 550 nm (Auclair and Voisin, 1985). The results are expressed as mean reduced NBT (relative to control—100%) ± SEM, from three separate determinations performed in duplicate.

#### Malondialdehyde (MDA)

Spectrophotometric method of Villacara et al. (1989) was used for determination of MDA concentration. MDA gives a red product after incubation with thiobarbituric acid (TBA) reagent (15% trichloroacetic acid and 0.375% TBA, water solution, Merck—Darmstadt, Germany), at 95°C and pH 3.5. Absorbance was measured at 532 nm. The results were expressed as mean MDA concentration (nmol/ml) ± SEM, from three separate determinations, performed in duplicate.

#### Total glutathione

DTNB-GSSG reductase recycling assay was used for determination of total glutathione content. The rate of formation of 5-thio-2-nitrobenzoic acid (TNBA), corresponding to total concentration of glutathione, was measured at 412 nm (Anderson, 1986). The results are expressed as mean concentration of glutathione (nmol/ml) ± SEM, from three separate measurements performed in duplicate.

#### Superoxide dismutase activity

Total SOD activity, which combines the activity of two SOD isoforms, cytoplasmic SOD1 (Cu,ZnSOD) and mitochondrial SOD2 (MnSOD), was evaluated by the epinephrine method. Activity of SOD (EC 1.15.1.1.) was assayed as inhibition of spontaneous autooxidation of epinephrine (Sigma-Aldrich—St. Louis, USA), by measuring absorbance at 480 nm. The kinetics of enzyme activity was followed in a carbonate buffer (50 mM, pH 10.2, containing 0.1 mM EDTA, Serva, Feinbiochemica—Heidelberg, New York), after the addition of 10 mM epinephrine and 5 mM KCN for MnSOD isoform (Sun and Zigman, 1978). The results were expressed as units per milligram of protein. One unit is defined as an amount of protein (enzyme) required for 50% of auto oxidation of epinephrine.

#### Catalase activity

CAT activity was determined by a method based on spectrophometric determination (405 nm) of colored complex formed between ammonium molibdate and H_2_O_2_ (Góth, 1991). Unit of CAT activity is defined as the amount of H_2_O_2_ reduced per minute (μmol H_2_O_2_/min). Data are expressed as mean CAT activity (units/mg protein) ± SEM; from three separate determinations performed in duplicate.

#### Glutathione peroxidase activity

Activity of GPx is measured by indirect spectrophotometric determination of the GPx -mediated NADPH consumption (340 nm), as previously described (Maral et al., 1977; Djukic et al., 2012). The results are expressed as miliunits per milligram of protein.

#### Glutathione reductase activity

Method for determining the activity of the GR is based on the ability of GR to catalyze the reduction of GSSG to GSH by the oxidation of the coenzyme NADPH to NADP^+^ (Freifelder, 1976). In the reaction as standard we used 100 mmol NAD^+^. The unit of enzyme activity is defined as number of micromols of NADPH oxidized per minute (μmol NADPH). The results were expressed as mean GR activity (mU/mg protein) ± SEM, from n separate determinations performed in duplicate.

### Measurement of intracellular ATP

Intracellular ATP was extracted from BV-2 cells with boiling water, as described previously (Yang et al., 2002). Medium was removed and boiling water was added to cells, which were quickly scraped to obtain cell suspensions. Cell suspensions were boiled for 10 min and centrifuged at 12,000 × g for 5 min, at 4°C. Supernatants were used for immediate determination of ATP by bioluminescent assay kit (Sigma Aldrich, St. Louis, USA), according to the manufacturer's instruction. Samples were incubated with the assay mix containing luciferin and luciferase and the luminescence intensity proportional to ATP content was measured with luminometer (CHAMELEON™V, Hidex, Turku, Finland). ATP standard curve was constructed for determination of ATP concentration in samples. Results are expressed as nmols of ATP per mg of protein.

### Statistical analysis

Results are expressed as mean values ± SEM. To assess statistical significance in all experiments, experiments were performed in duplicate or triplicate determinations using three separate cell preparations. Statistical analysis was completed with GraphPad Prism software. Data were analyzed using One-Way ANOVA (except data for ATP, which were analyzed using Two-Way ANOVA) with Bonferroni *post-hoc* analysis. Values of *p* < 0.05 were considered statistically significant.

## Results

### Benfotiamine suppresses LPS-induced CD40 expression in BV-2 cells

**Figure 1:**
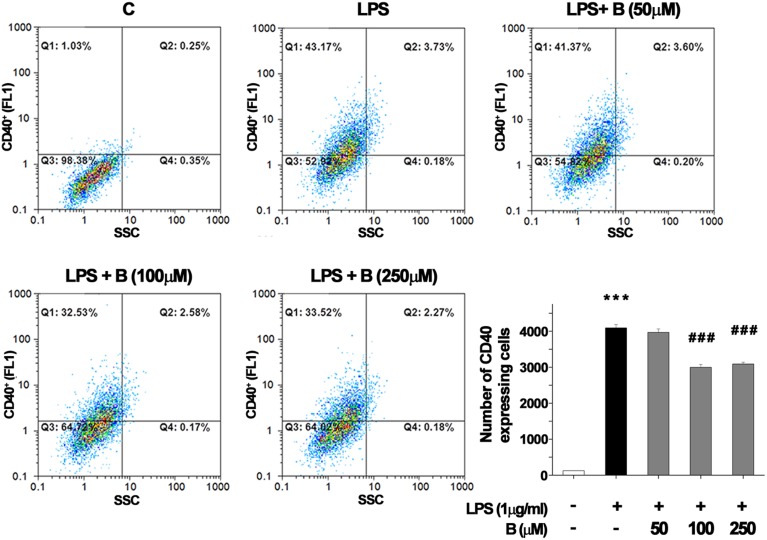
**FACS analysis of CD40 expression in BV-2 cells**. Expression of immunoregulatory receptor CD40 was analyzed with FACS and representative dot plots are shown for control (C), group treated with LPS (LPS) for 24 h, and groups pre treated with benfotiamine in doses of 50, 100, and 250 μM and then treated with LPS for 24 h [LPS + B (50 μM), LPS + B (100 μM), and LPS + B (250 μM)]. Statistical analysis was performed and mean values from three independent experiments are presented on the graph, for control cultures (white bar), LPS treated cells (black bar) and groups pre treated with different doses of benfotiamine and then stimulated with LPS (gray bars). ^***^p < 0.001 compared with control group, ^*###*^*p* < 0.001 compared with LPS treated group.

CD40 expression by BV-2 cells was used as an indicator of microglial activation (Qin et al., 2005). In control BV-2 cells constitutive expression of surface CD40 was low, and only about 100 per 10,000 cells analyzed by FACS expressed CD40 receptor (Figure 1). Treatment with LPS (1 μg/ml) for 24 h upregulated the expression of CD40 by approximately 40% (4000 cells were positive for CD40). Pretreatment of BV-2 cells with 100 and 250 μM benfotiamine significantly decreased the number of CD40 expressing cells to 3000 (*p* < 0.001). The results indicate that benfotiamine influence on activated BV-2 cells involves expression of CD40.

### Benfotiamine does not affect viability of LPS-activated BV-2 cells

**Figure 2:**
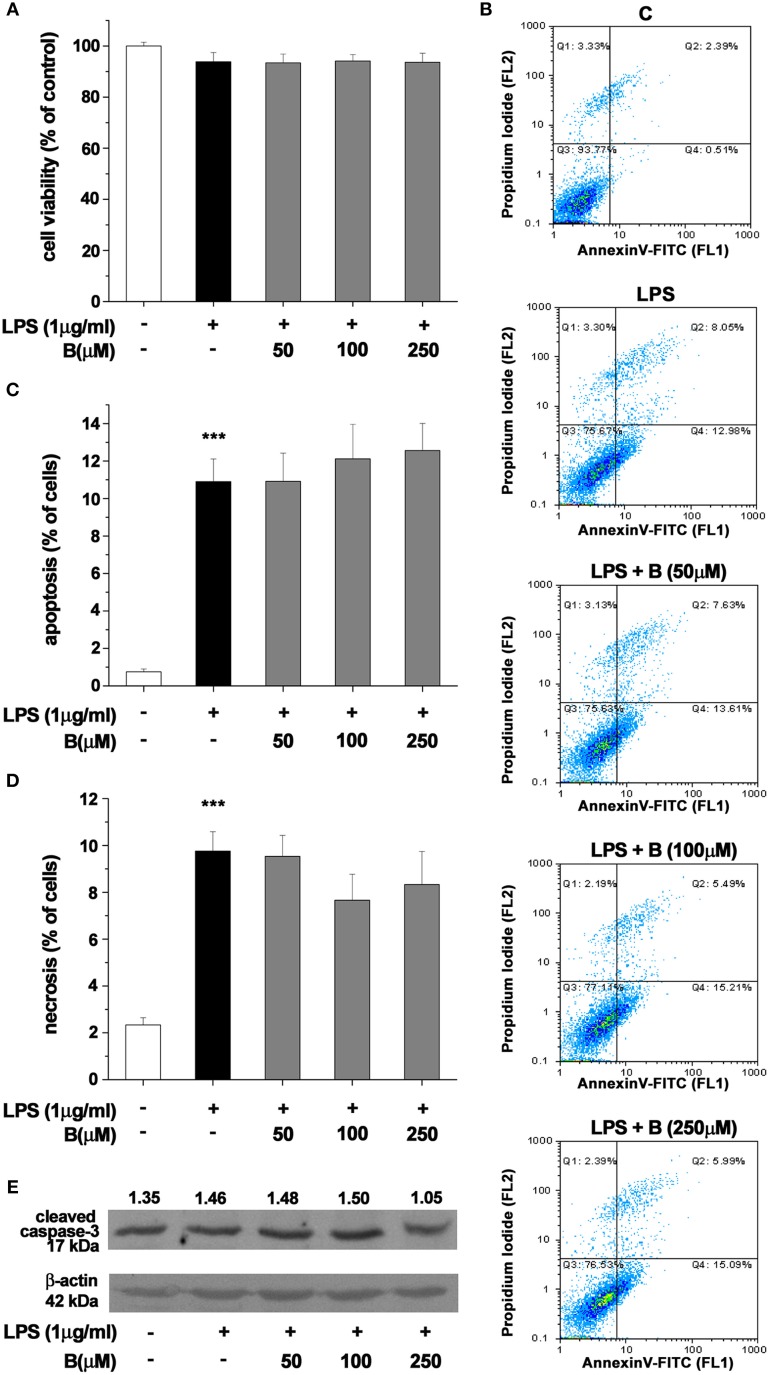
**Effect of benfotiamine on viability and apoptotic and necrotic cell death of LPS (1 μg/ml) stimulated BV-2 cells**. Cell viability was evaluated with MTT assay, 24 h after treatment with LPS **(A)**. FACS analysis of apoptotic and necrotic cell death was performed with FITC labeled annexin V and propidium iodide **(B)**. Apoptotic cells were labeled with annexin V, whereas necrotic cells were positive for both annexin V and propidium iodide. Percentage of apoptotic **(C)** and necrotic **(D)** cells was determined from three independent cell preparations. ^***^p < 0.001 compared with control group. Activation of caspase—3 was assessed with western blotting for active caspase—3 fragment **(E)**. Representative blot from three independent experiments is shown. Band intensity was analyzed, compared to β-actin of the same lane and results are expressed in arbitrary units.

Effect of benfotiamine pretreatment on BV-2 cells' state was determined using MTT assay and Annexin V/propidium iodide FACS analysis after 24 h treatment with LPS (1 μg/ml. Benfotiamine did not change total mitochondrial activity of LPS-stimulated BV-2 cells, as deduced from stable MTT values (Figure 2A). Annexin V/PI FACS analysis (Figure 2B) demonstrated, however, that LPS caused modest increase in the proportion of apoptotic (about 11% of cells, Figures 2B,C) and necrotic (approximately 10% of cells, Figures 2B,D) cells in cultures. Importantly, benfotiamine did not significantly affect the viability of LPS-stimulated cells (Figure 2). The finding was further substantiated by immunoblot analysis of active fragment of caspase-3. A slight increase in caspase-3 expression was observed in LPS stimulated cells, whereas benfotiamine showed no such effects (Figure 2E). Furthermore, the treatments did not affect cell proliferation (Figure S2).

### Benfotiamine decreases production of NO, ^·^O^−^_2_, and MDA

**Figure 3:**
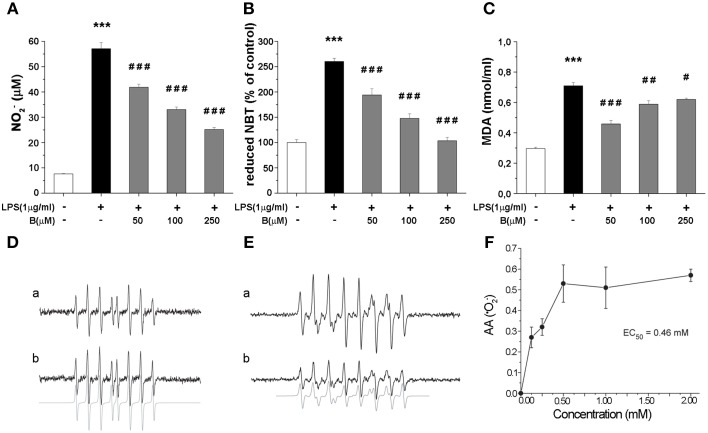
**Effect of benfotiamine on levels of NO, ^·^O^−^_2_ and MDA in BV-2 cells stimulated with LPS (1 μg/ml) for 24 h. (A)** NO production was measured with Griess assay 24 h after LPS treatment and results are expressed as mean values ± SEM (*n* = 3). Intracellular concentrations of ^·^O^−^_2_ **(B)** and MDA **(C)** were measured in three independent cell preparations of BV-2 cells after 24 h treatment with LPS. **(D)** EPR signals of DEPMPO/OH adducts in the Fenton system without (a) or with (b) benfotiamine (1 mM); **(E)** Characteristic EPR signals of DEPMPO/OOH generated by SOTS-1 without (a) or with (b) benfotiamine (0.5 mM) **(F)** Antioxidative activity of benfotiamine against ^·^O^−^_2_. ^***^p < 0.001 compared with control group, ^#^*p* < 0.05, ^*##*^*p* < 0.01, ^*###*^*p* < 0.001 compared with LPS treated group.

Production of NO and intracellular content of superoxide anions (^·^O^−^_2_) and MDA were determined as they represent critical indicators of oxidative stress. LPS stimulation (1 μg/ml, 24 h) resulted in several fold increase of levels of NO, ^·^O^−^_2_ and MDA (Figures 3A–C). Benfotiamine pretreatment (30 min prior to LPS) suppressed NO release (Figure 3A) in a dose-dependent manner (*p* < 0.001). Concentration of ^·^O^−^_2_, was increased 2.5-fold with LPS stimulation (Figure 3B). This was suppressed by pretreatment with benfotiamine, which, at 250 μM dose returned ^·^O^−^_2_ content to control levels (*p* < 0.001). Furthermore, benfotiamine substantially downgraded LPS-induced lipid peroxidation (Figure 3C) at all examined doses. In the presence of benfotiamine the yield of DEPMPO/OH adduct was slightly increased (about 10%) compared to control system (Figure 3D). This implies a modest pro-oxidative activity of benfotiamine, probably via Fe^3+^ reduction. On the other hand, benfotiamine significantly affected generation of ^·^O^−^_2_ (Figure 3E) at concentrations corresponding to those applied in experiments on microglia (Figure 3F). It was estimated that the rate constant for the reaction between benfotiamine and ^·^O^−^_2_ was 68 ± 19 M^−1^ s^−1^. These data together imply that benfotiamine exhibits significant antioxidative activity in BV-2 cells.

### Benfotiamine modulates expression of enzymes involved in antioxidative defense

**Figure 4:**
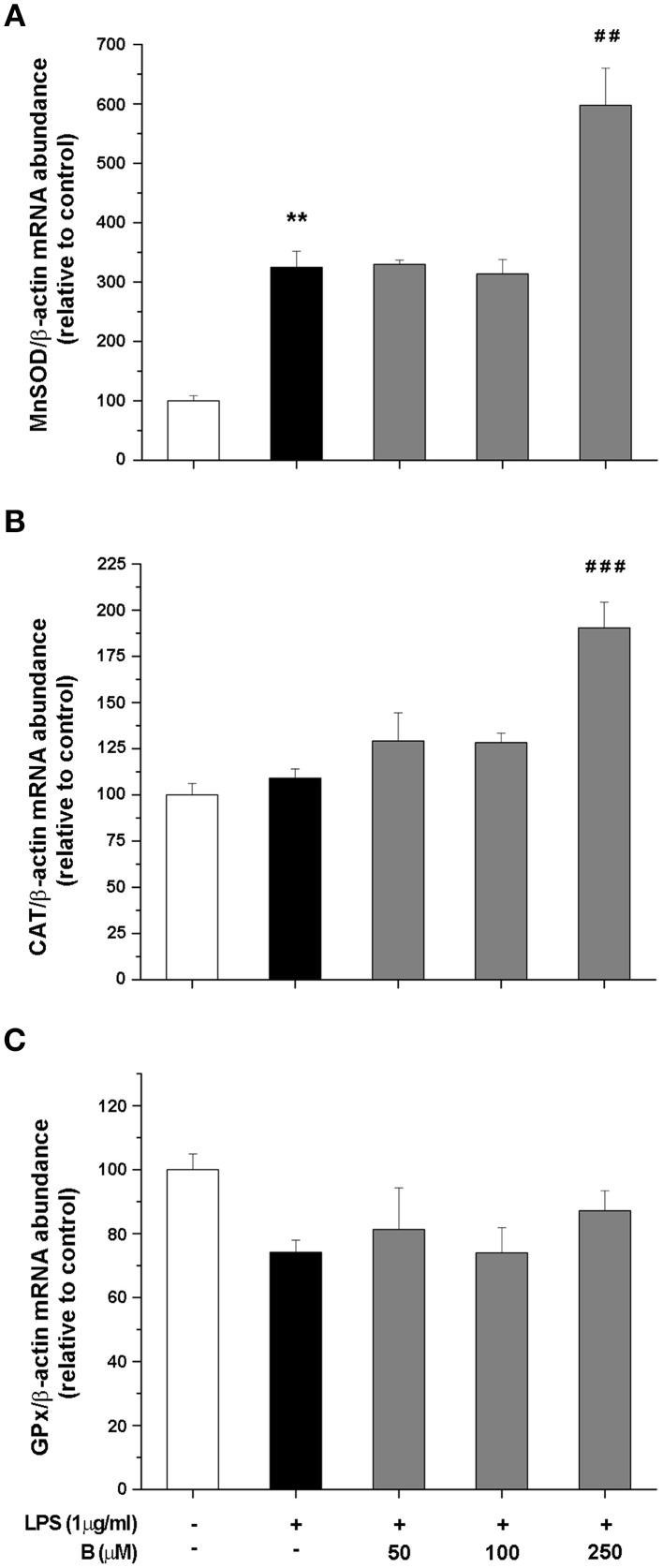
**Effect of benfotiamine on gene expression of antioxidative enzymes in LPS stimulated BV-2 cells**. Expression of MnSOD **(A)**, CAT **(B)**, and GPx **(C)** was evaluated with RT PCR and expressed relative to the expression of β-actin mRNA. BV-2 cells were pretreated with different doses of benfotiamine (50, 100, and 250 μM) for 30 min, stimulated with LPS for 4 h and then harvested for mRNA isolation. Three independent experiments were performed and statistical significance was marked as ^**^p < 0.01 compared with control group, ^*##*^*p* < 0.01, ^*###*^*p* < 0.001 compared with LPS treated group.

To shed more light on possible mechanism of antioxidative actions of benfotiamine, we further determined gene expression levels for SOD2, CAT, and GPx by qRT-PCR. These enzymes constitute essential part of antioxidative cellular defense, since SOD2 dismutates ^·^O^−^_2_ to H_2_O_2_, while CAT and GPx convert H_2_O_2_ to water. BV-2 cells were pretreated with benfotiamine and the mRNA content was determined 4 h after LPS stimulation (Figure 4). SOD2 gene expression was promoted three-fold by LPS, while benfotiamine, at its highest concentration, induced even higher increase (*p* < 0.01, Figure 4A). On the other hand, while LPS alone did not affect expression of CAT gene, 250 μM benfotiamine induced 2-fold increase in the CAT-mRNA abundance compared to LPS group (*p* < 0.001, Figure 4B). Finally, no significant effects on GPx-mRNA were observed (Figure 4C). Taken together, these results suggest that antioxidative actions of benfotiamine may be partly mediated via induction of antioxidative enzyme genes, including SOD2 and CAT.

### Benfotiamine upregulates protein expression of antioxidative defense enzymes

**Figure 5:**
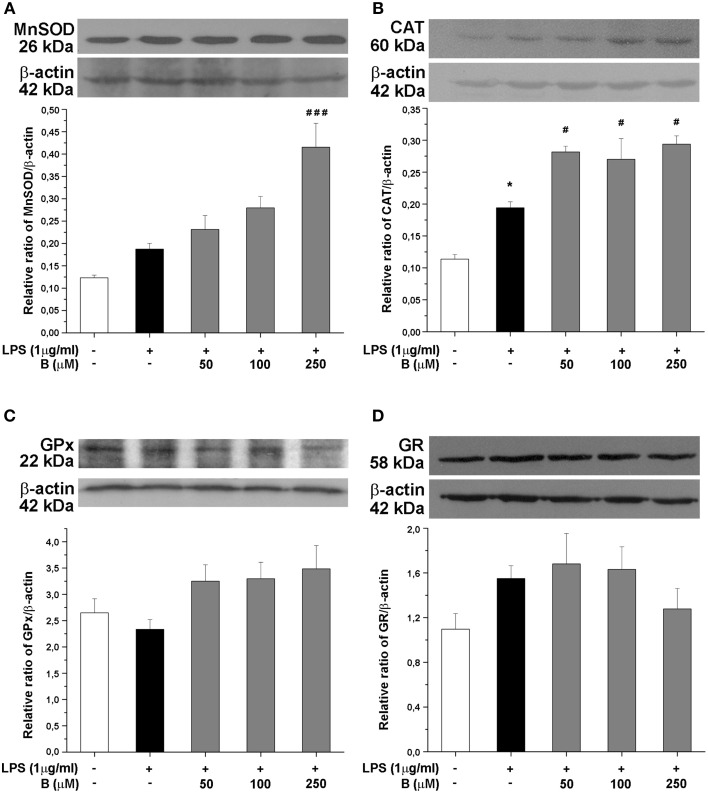
**Effect of benfotiamine on protein expression of antioxidative enzymes in LPS treated BV-2 cells**. Protein expression of MnSOD **(A)**, CAT **(B)**, GPx **(C)**, and GR **(D)** was evaluated with western blotting, 24 h after LPS treatment. Representative blots from three independent experiments are shown. Protein bands were analyzed with Image Quant 5.2 software, compared to β-actin of the same lane and results in graphs are expressed as percentage of control group. ^*^P < 0.05 compared with control group, ^#^*p* < 0.05, ^*###*^*p* < 0.001 compared with LPS treated group.

Expression of antioxidative enzymes SOD2, CAT, GPx, and GR was further evaluated at the protein level by Western blot analysis (Figure 5). Among the antioxidative enzymes tested, LPS affected only protein expression of CAT (Figure 5B). Benfotiamine, however, induced significant effects on SOD2 (*p* < 0.001, Figure 5A) and CAT protein expression (*p* < 0.05, Figure 5B). No changes in the protein expression of GPx and GR were observed (Figures 5C,D, respectively).

### Benfotiamine enhances the activity of antioxidative enzymes and increases glutathione content in BV-2 cells

**Figure 6:**
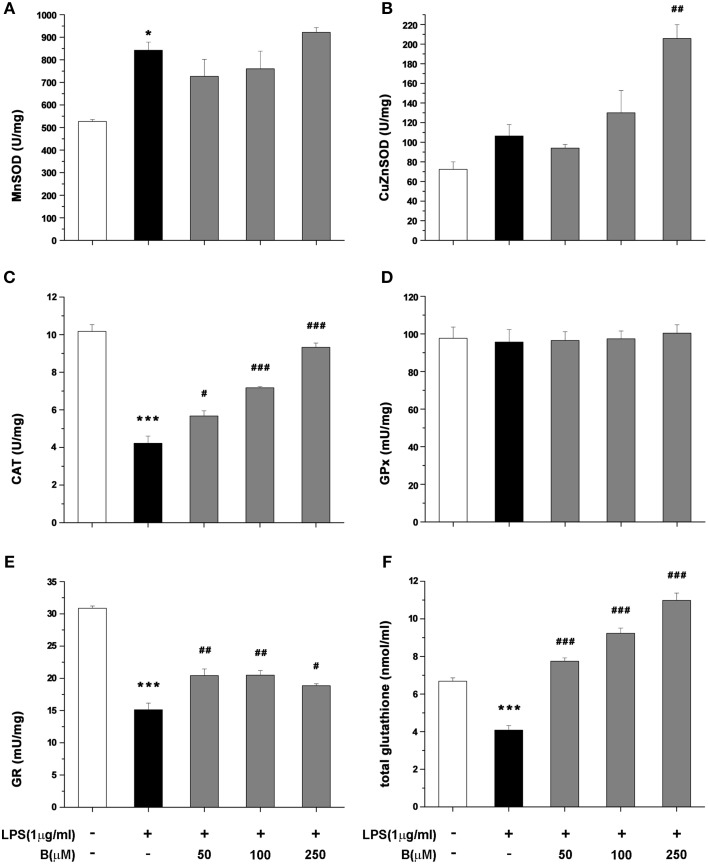
**Effect of benfotiamine on activity of antioxidative enzymes and total glutathione content in LPS treated BV-2 cells**. Activity of MnSOD **(A)**, Cu,ZnSOD **(B)**, CAT **(C)**, GPx **(D)**, GR **(E)**, and total glutathione content **(F)** was analyzed in BV-2 cells following LPS treatment for 24 h. The results of activity of antioxidative enzymes are expressed as mean specific activities (U/mg) ± SEM from three independent cell preparations. ^*^p < 0.05, ^***^p < 0.001 compared with control group, ^#^*p* < 0.05, ^*##*^*p* < 0.01, ^*###*^*p* < 0.001 compared with LPS treated group.

Antioxidative potential of benfotiamine in BV-2 cells was further analyzed in terms of antioxidative enzymes activity and intracellular content of total glutathione, main non-enzymatic antioxidant in microglia. The cells were pretreated with benfotiamine in 50, 100, and 250 μM dose for 30 min and then stimulated with LPS (1 μg/ml) for 24 h. Activity of MnSOD increased upon LPS stimulation, whereas it was not affected by benfotiamine (Figure 6A). On the contrary, activity of Cu,ZnSOD was not affected by LPS while it was doubled by pretreatment with 250 μM benfotiamine (*p* < 0.01, Figure 6B). CAT activity (Figure 6C) was substantially inhibited in cells treated with LPS (from 10 U/mg in control cells to 4 U/mg in LPS treated group, *p* < 0.001). Benfotiamine induced dose-dependent increase reaching the control CAT activity at the highest concentration (approximately 9 U/mg, *p* < 0.001). Benfotiamine had no effect on CAT activity in non-stimulated cells (Figure S1A). The inhibition of iNOS ameliorated the inhibitory effects of LPS stimulation on CAT activity (Figure S1B). GPx activity was not affected by LPS stimulation nor benfotiamine treatment (Figure 6D). Activity of GR decreased after LPS stimulation and slightly increased after the benfotiamine pretreatments (Figure 6E). Finally, total glutathione content was substantially decreased in the microglial cells stimulated with LPS (Figure 6F). This was annihilated by benfotiamine which provoked a significant increase compared to control values.

### Benfotiamine increases intracellular ATP content

**Figure 7:**
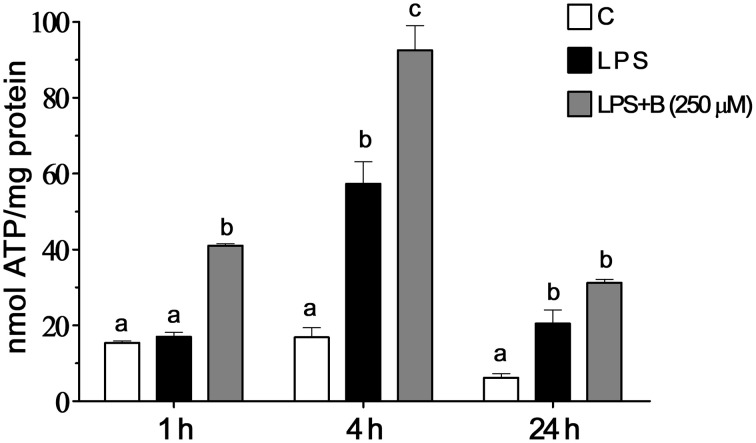
**Effect of benfotiamine on intracellular ATP content of LPS stimulated BV-2 cells**. Concentration of ATP were determined in control groups (white bars), cells treated with LPS (black bars) and cells pre-treated with 250 μM benfotiamine and then stimulated with LPS for 1, 4, and 24 h (gray bars). The results are expressed in nmol per mg of protein and represent mean values from three independent experiments ± SEM. The groups not sharing a common letter are significantly different (*p* < 0.05).

Intracellular ATP level was measured in BV-2 cells at three time points (1, 4, and 24 h) after LPS stimulation (Figure 7). In cells treated with LPS, intracellular ATP content was higher compared to control levels 4 h after the treatment and remained higher 24 h later. Pretreatment with 250 μM benfotiamine induced an increase of ATP content at 1 h after the LPS stimulation. Increased level remained increased 24 h later. The effects of LPS and benfotiamine peaked at 4 h.

## Discussion

Activated microglia undergoes transformation which involves morphological changes, induction of surface markers, increased production of various proinflammatory cytokines and acquisition of cellular profile that generates oxidative burst (Block and Hong, 2005; Bordt and Polster, 2014). Pertinent to the latter, activated microglia releases ROS and RNS, such as ^·^O^−^_2_ and NO (as shown here) via NADPH oxidase activity (on membrane) and inducible NO synthase—iNOS (intracellular), respectively (Dringen, 2005). Such setup limits the production of highly dangerous peroxynitrite (^·^O^−^_2_ + NO → ONOO^−^) in the extracellular fluid, thus targeting outer targets (such as pathogens), but simultaneously protecting microglia from self-inflicting damage. Self-protection of activated microglia is also effectuated by significant induction of intrinsic antioxidative system. Our data confirm increased expression of CAT and SOD2 and increased SOD2 activity in LPS activated microglia which is in agreement with previously reported data (Dringen, 2005). Increased expression of CAT contribute to the efficient removal of H_2_O_2_, which permeates cell membrane, after being produced by extracellular dismutation of ^·^O^−^_2_ (Gao et al., 2003). The observed increase in the level of mRNA and enzymatic activity of SOD2 is most likely related to increased mitochondrial activity (and number) in activated microglia (Park et al., 2013). Increased demands for energy production in LPS activated microglia is met through enhanced ATP synthesis which requires the acceleration of electron transfer chain. Under such conditions, electron leak and ^·^O^−^_2_ generation in mitochondria are promoted and this is mitigated through higher activity of SOD2 (Bordt and Polster, 2014). Increased SOD1 activity (this SOD is mainly located in the cytosol), might be a response to increased cyclooxygenase-2 activity in activated microglia (Siomek, 2012). This enzyme has ^·^O^−^_2_ as a by-product (Marnett et al., 1999). Interestingly, CAT showed increased levels but lower activities in activated microglia compared to resting cells. This implies inhibition. Pertinent to this, CAT is reversibly inhibited by NO (Brown, 1995), whereas GR is inhibited by ONOO^−^ (Francescutti et al., 1996).

Benfotiamine modulates oxidative activity but does not kill microglia, which is a preferred way of action for drugs targeting hyper-active microglia in neurological conditions (Uttara et al., 2009; Luo et al., 2010; Roth et al., 2014). Thiamine, a benfotiamine metabolite, enters the CNS, as shown using high performance liquid chromatography which has demonstrated higher thiamine concentration in the brain after oral administration of benfotiamine in mice. Beneficial effects of benfotiamine on mouse model of Alzheimer's disease have been attributed to both benfotiamine and thiamine (Pan et al., 2010). Benfotiamine decreases the expression of CD40, a protein that determines antigen presenting ability of microglia and activation of NF-κB signaling (Kim et al., 2002; Kraft and Harry, 2011; Morgan and Liu, 2011). CD40 has an important role in neuroinflammatory diseases and abnormal expression of CD40 and its ligand CD154 has been shown in Alzheimer's disease (Calingasan et al., 2002; Giunta et al., 2010), multiple sclerosis (Gerritse et al., 1996), and HIV-1 associated dementia (D'Aversa et al., 2002). Considering that activation of CD40 receptor in microglia leads to expression of iNOS and production of TNF-α (Jana et al., 2001, 2002) and other proinflammatory molecules (Chen et al., 2006), benfotiamine's ability to suppress CD40 expression can alleviate inflammation in neurological disorders. Benfotiamine also exhibits strong antioxidative abilities and suppresses oxidative burst. It decreased NO and ^·^O^−^_2_ production and lipid peroxidation of microglial membrane. The potency of benfotiamine to inhibit lipid peroxidation and ^·^O^−^_2_ overproduction has been observed previously in murine kidney exposed to cisplatin and endothelium exposed to nicotine (Balakumar et al., 2008; Harisa, 2013). Benfotiamine induced fall in NO production is most likely caused by down-regulation of iNOS expression due to suppressed NFκB signaling in benfotiamine-treated microglia (Bozic et al., 2015). The effects on ^·^O^−^_2_ production might be based upon the capacity of benfotiamine to directly scavenge this radical, but also on its inhibitory effects on the activity of NF-κB, which is involved in the expression of NADPH oxidase (Morgan and Liu, 2011; Siomek, 2012). However, the most important finding here is that benfotiamine upregulated the intracellular antioxidative system, thus increasing the capacity of activated microglia to buffer the excessive production of ROS.

Namely, in the presence of benfotiamine, microglial cells showed increased levels of CAT and SOD2 mRNA, increased amounts of CAT and SOD2, and increased activity of CAT, GR, and SOD1. Benfotiamine-provoked increase of CAT activity showed dose-dependency. The effect is reciprocally proportional to the level of NO production. Hence, increased CAT activity can be attributed to benfotiamine-provoked decrease in the production of NO (CAT inhibitor), as well as to the stimulation of CAT expression. Of note, in the previous report we have applied a less sensitive Griess protocol (no NO^−^_3_ reduction) which allowed for benfotiamine-provoked suppression of NO to be observed, but dose-dependency was unnoticed (Bozic et al., 2015). The level of GR was not significantly increased in benfotiamine-treated cells, but its activity was increased. This implies that benfotiamine ameliorated the inhibition of GR, which is provoked by NO derivative—ONOO^−^. The production of both, NO and ONOO^−^ in activated microglia are based on iNOS activity (Kumar et al., 2014; Bozic et al., 2015). It is important to point out that NO and ROS production are intertwined. For example, H_2_O_2_ activates NF-κB activity and hence promotes the expression of iNOS (Andrades et al., 2011). It appears that benfotiamine might initiate a feedback loop that has the silencing of pro-oxidative activity of activated microglia as a result. In brief, benfotiamine provokes an increase in the level of H_2_O_2_-removing enzyme—CAT, which should result in lower NFκB activity and iNOS levels, as we reported previously (Bozic et al., 2015). This further leads to lower NO levels, and to de-inhibition of CAT. This is implied by the fact that iNOS inhibition by L-NAME led to increased CAT activity in LPS stimulated cells. Of note, peroxynitrite irreversibly inhibits GR, which most likely accounts for the modest effects of benfotiamine on this enzyme as compared to the effects on CAT activity. Finally, this loop might be involved in benfotiamine-provoked suppression of microglia activation (lower CD40, proinflammatory mediators, such as TNF-α and IL-6), since it is known that ROS can amplify microglia inflammatory response (Mander et al., 2006).

Increased SOD2 expression and ATP levels imply that benfotiamine promotes mitochondrial activity/number. One potential mechanism is that benfotiamine activates some xenobiotic-like response. The removal of xenobiotics requires energy, and they have been shown to promote expression of SOD2 (Curtis et al., 2007). In addition, benfotiamine upregulated glutathione system. The total glutathione was increased 2- to 3-fold in activated microglia exposed to benfotiamine. This major change may only come from *de novo* synthesis of glutathione (Lu, 2009). Such response is common in handling xenobiotics and reactive molecules in CNS (Valdovinos-Flores and Gonsebatt, 2012; Zhang et al., 2013). Benfotiamine actions fall under a relatively novel strategy in antioxidative therapy which employs hormesis i.e., exposure to one stressor increases resistance to another stressor (Gems and Partridge, 2008). Namely, drugs, such as dimethyl fumarate or ethyl pyruvate (Wilms et al., 2010; Miljkovic et al., 2015), or natural products, such as polyphenols and other mildly stressful compounds (Talalay et al., 2003; Moskaug et al., 2005) activate antioxidative system, which then protects the cell from oxidative stress that is inflicted by other sources/processes. In the present case, benfotiamine-mediated stimulation of antioxidative system is more important for increasing the capacity of microglia to buffer oxidative burst than for protecting microglia *per se*. In conditions where no real threat (such as infection agents) is present, and microglia enters hyper-reactive state as a side-reaction to some pathological processes, benfotiamine might silence pro-oxidative activity of microglia to alleviate/prevent oxidative damage on neighboring CNS cells.

Our results open the possibility for benfotiamine application in neurodegenerative conditions which show hyper-reactive microglia, such as Alzheimer's, Parkinson's disease, amyotrophic lateral sclerosis or multiple sclerosis. Further research on animal model studies are warrant in order to evaluate benfotiamine capacity to mitigate the microglial component of pathology of neurological diseases.

## Funding

This work was supported by the Ministry of Education, Science and Technological Development of the Republic of Serbia, Project No. III41014.

## Author contributions

Conceived and designed the experiments: IL, IB, NN. Performed the experiments: IB, DS, IS. Analyzed the data: IL, IB, DS, SP. Contributed to the writing of the manuscript: IL, IB, NN, SP.

### Conflict of interest statement

The authors declare that the research was conducted in the absence of any commercial or financial relationships that could be construed as a potential conflict of interest.

## Supplementary material

Figure S1 **CAT activity—effect of benfotiamine in basal conditions and effect of iNOS inhibitor (L-NAME).** Activity of CAT was determined 24 h after treatment with benfotiamine (50, 100 and 250 μM) in the absence of LPS stimulation **(A)**. CAT activity was examined in BV-2 cells treated with L-NAME (500 μM) for 1 h and then stimulated with LPS for 24 h **(B)**. The results are expressed as mean specific activities (U/mg) ± SEM from three independent cell preparations. ^***^*p* < 0.001 compared with control group, ^###^*p* < 0.001 compared with LPS treated group.

Figure S2 **Effect of benfotiamine and LPS treatment on BV-2 cell proliferation.** BV-2 cells were treated with benfotiamine (250 μM), LPS (1 μg/ml) or their combination for 24 h, stained with Ki-67 antibody and analyzed with FACS. Representative dot plots are shown. Statistical analysis was performed and mean values from three independent experiments are presented on the graph.

## References

[B1] Aguzzi A. Barres B. A. Bennett M. L. (2013). Microglia: scapegoat, saboteur, or something else? Science 339, 156–161. 10.1126/science.1227901 23307732 PMC4431634

[B2] Andersen J. K. (2004). Oxidative stress in neurodegeneration: cause or consequence? Nat. Rev. Neurosci. 5, S18–S25. 10.1038/nrn1434 15298006

[B3] Anderson M. E. (1986). Tissue glutathione, in Handbook of Methods for Oxygen Radical Research, ed Greenwald R. A. (Boca Raton, FL: CRC Press), 317–323.

[B4] Andrades M. É. Morina A. Spasic S. Spasojevic I. (2011). Bench-to-bedside review: sepsis - from the redox point of view. Crit. Care 15, 230. 10.1186/cc10334 21996422 PMC3334726

[B5] Auclair C. Voisin E. (1985). Nitroblue tetrazolium reduction, in Handbook of Methods for Oxygen Radical Research, ed Greenwald R. A. (Boca Raton, FL: CRC Press), 123–132.

[B6] Balakumar P. Rohilla A. Krishan P. Solairaj P. Thangathirupathi A. (2010). The multifaceted therapeutic potential of benfotiamine. Pharmacol. Res. 61, 482–488. 10.1016/j.phrs.2010.02.008 20188835

[B7] Balakumar P. Sharma R. Singh M. (2008). Benfotiamine attenuates nicotine and uric acid-induced vascular endothelial dysfunction in the rat. Pharmacol. Res. 58, 356–363. 10.1016/j.phrs.2008.09.012 18951979

[B8] Barger S. W. Goodwin M. E. Porter M. M. Beggs M. L. (2007). Glutamate release from activated microglia requires the oxidative burst and lipid peroxidation. J. Neurochem. 101, 1205–1213. 10.1111/j.1471-4159.2007.04487.x 17403030 PMC1949347

[B9] Barnham K. J. Masters C. L. Bush A. I. (2004). Neurodegenerative diseases and oxidative stress. Nat. Rev. Drug. Discov. 3, 205–214. 10.1038/nrd1330 15031734

[B10] Bitsch R. Wolf M. Möller J. Heuzeroth L. Grüneklee D. (1991). Bioavailability assessment of the lipophilic benfotiamine as compared to a water-soluble thiamin derivative. Ann. Nutr. Metab. 35, 292–296. 10.1159/000177659 1776825

[B11] Blasi E. Barluzzi R. Bocchini V. Mazzolla R. Bistoni F. (1990). Immortalization of murine microglial cells by a v-raf/v-myc carrying retrovirus. J. Neuroimmunol. 27, 229–237. 10.1016/0165-5728(90)90073-V 2110186

[B12] Block M. L. Hong J. S. (2005). Microglia and inflammation-mediated neurodegeneration: multiple triggers with a common mechanism. Prog. Neurobiol. 76, 77–98. 10.1016/j.pneurobio.2005.06.004 16081203

[B13] Block M. L. Zecca L. Hong J. S. (2007). Microglia-mediated neurotoxicity: uncovering the molecular mechanisms. Nat. Rev. Neurosci. 8, 57–69. 10.1038/nrn2038 17180163

[B14] Bordt E. A. Polster B. M. (2014). NADPH oxidase- and mitochondria-derived reactive oxygen species in proinflammatory microglial activation: a bipartisan affair? Free Radic. Biol. Med. 76, 34–46. 10.1016/j.freeradbiomed.2014.07.033 25091898 PMC4252610

[B15] Bozic I. Savic D. Laketa D. Bjelobaba I. Milenkovic I. Pekovic S.. (2015). Benfotiamine attenuates inflammatory response in LPS stimulated BV-2 microglia. PLoS ONE 10:e0118372. 10.1371/journal.pone.0118372 25695433 PMC4335016

[B16] Brown G. C. (1995). Reversible binding and inhibition of catalase by nitric oxide. Eur. J. Biochem. 232, 188–191. 10.1111/j.1432-1033.1995.tb20798.x 7556149

[B17] Calingasan N. Y. Erdely H. A. Altar C. A. (2002). Identification of CD40 ligand in Alzheimer's disease and in animal models of Alzheimer's disease and brain injury. Neurobiol. Aging 23, 31–39. 10.1016/S0197-4580(01)00246-9 11755016

[B18] Ceylan-Isik A. F. Wu S. Li Q. Li S. Y. Ren J. (2006). High-dose benfotiamine rescues cardiomyocyte contractile dysfunction in streptozotocin-induced diabetes mellitus. J. Appl. Physiol. 1, 150–156. 10.1152/japplphysiol.00988.2005 16166234

[B19] Chen K. Huang J. Gong W. Zhang L. Yu P. Wang J. M. (2006). CD40/CD40L dyad in the inflammatory and immune responses in the central nervous system. Cell. Mol. Immunol. 3, 163–169. 16893496

[B20] Curtis C. Landis G. N. Folk D. Wehr N. B. Hoe N. Waskar M.. (2007). Transcriptional profiling of MnSOD-mediated lifespan extension in Drosophila reveals a species-general network of aging and metabolic genes. Genome Biol. 8:R262. 10.1186/gb-2007-8-12-r262 18067683 PMC2246264

[B21] D'Aversa T. G. Weidenheim K. M. Berman J. W. (2002). CD40-CD40L interactions induce chemokine expression by human microglia: implications for human immunodeficiency virus encephalitis and multiple sclerosis. Am. J. Pathol. 160, 559–567. 10.1016/S0002-9440(10)64875-4 11839576 PMC1850648

[B22] Djukic M. M. Jovanovic M. D. Ninkovic M. Stevanovic I. Ilic K. Curcic M.. (2012). Protective role of glutathione reductase in paraquat induced neurotoxicity. Chem. Biol. Interact. 199, 74–86. 10.1016/j.cbi.2012.05.008 22721943

[B23] Dringen R. (2005). Oxidative and antioxidative potential of brain microglial cells. Antioxid. Redox Signal. 7, 1223–1233. 10.1089/ars.2005.7.1223 16115027

[B24] Frakes A. E. Ferraiuolo L. Haidet-Phillips A. M. Schmelzer L. Braun L. Miranda C. J.. (2014). Microglia induce motor neuron death via the classical NF-κB pathway in amyotrophic lateral sclerosis. Neuron 81, 1009–1023. 10.1016/j.neuron.2014.01.013 24607225 PMC3978641

[B25] Francescutti D. Baldwin J. Lee L. Mutus B. (1996). Peroxynitrite modification of glutathione reductase: modeling studies and kinetic evidence suggest the modification of tyrosines at the glutathione disulfide binding site. Protein Eng. 9, 189–194. 10.1093/protein/9.2.189 9005440

[B26] Freifelder D. (1976). Physical Biochemistry. New York, NY: W. H. Freeman and Company.

[B27] Fujiwara M. (1954). Allithiamine: a newly found derivative of vitamin B. J. Biochem. 2, 273–285.

[B28] Gao H. M. Hong J. S. Zhang W. Liu B. (2003). Synergistic dopaminergic neurotoxicity of the pesticide rotenone and inflammogen lipopolysaccharide: relevance to the etiology of Parkinson's disease. J. Neurosci. 23, 1228–1236. 12598611 10.1523/JNEUROSCI.23-04-01228.2003 PMC6742266

[B29] Gems D. Partridge L. (2008). Stress-response hormesis and aging: “that which does not kill us makes us stronger.” Cell Metab. 7, 200–203. 10.1016/j.cmet.2008.01.001 18316025

[B30] Gerritse K. Laman J. D. Noelle R. J. Aruffo A. Ledbetter J. A. Boersma W. J.. (1996). CD40-CD40 ligand interactions in experimental allergic encephalomyelitis and multiple sclerosis. Proc. Natl. Acad. Sci. U.S.A. 93, 2499–2504. 10.1073/pnas.93.6.2499 8637903 PMC39826

[B31] Gertig U. Hanisch U. K. (2014). Microglial diversity by responses and responders. Front. Cell Neurosci. 8:101. 10.3389/fncel.2014.00101 24744702 PMC3978327

[B32] Giunta B. Rezai-Zadeh K. Tan J. (2010). Impact of the CD40-CD40L dyad in Alzheimer's disease. CNS Neurol. Disord. Drug Targets 9, 149–155. 10.2174/187152710791012099 20205645 PMC2892111

[B33] Giunti D. Parodi B. Cordano C. Uccelli A. Kerlero de Rosbo N. (2014). Can we switch microglia's phenotype to foster neuroprotection? Focus on multiple sclerosis. Immunology 141, 328–339. 10.1111/imm.12177 24116890 PMC3930371

[B34] González H. Elgueta D. Montoya A. Pacheco R. (2014). Neuroimmune regulation of microglial activity involved in neuroinflammation and neurodegenerative diseases. J. Neuroimmunol. 274, 1–13. 10.1016/j.jneuroim.2014.07.012 25091432

[B35] Góth L. (1991). A simple method for determination of serum catalase activity and revision of reference range. Clin. Chim. Acta 196, 143–151. 10.1016/0009-8981(91)90067-M 2029780

[B36] Hammes H. P. Du X. Edelstein D. Taguchi T. Matsumura T. Ju Q.. (2003). Benfotiamine blocks three major pathways of hyperglycemic damage and prevents experimental diabetic retinopathy. Nat. Med. 9, 294–299. 10.1038/nm834 12592403

[B37] Harisa G. I. (2013). Benfotiamine enhances antioxidant defenses and protects against cisplatin-induced DNA damage in nephrotoxic rats. J. Biochem. Mol. Toxicol. 27, 398–405. 10.1002/jbt.21501 23716490

[B38] Henn A. Lund S. Hedtjärn M. Schrattenholz A. Pörzgen P. Leist M. (2009). The suitability of BV2 cells as alternative model system for primary microglia cultures or for animal experiments examining brain inflammation. ALTEX 26, 83–94. 19565166 10.14573/altex.2009.2.83

[B39] Jana M. Dasgupta S. Liu X. Pahan K. (2002). Regulation of tumor necrosis factor-alpha expression by CD40 ligation in BV-2 microglial cells. J. Neurochem. 80, 197–206. 10.1046/j.0022-3042.2001.00691.x 11796758

[B40] Jana M. Liu X. Koka S. Ghosh S. Petro T. M. Pahan K. (2001). Ligation of CD40 stimulates the induction of nitric-oxide synthase in microglial cells. J. Biol. Chem. 276, 44527–44533. 10.1074/jbc.M106771200 11551948 PMC2041871

[B41] Jekabsone A. Mander P. K. Tickler A. Sharpe M. Brown G. C. (2006). Fibrillar beta-amyloid peptide Abeta1-40 activates microglial proliferation via stimulating TNF-alpha release and H2O2 derived from NADPH oxidase: a cell culture study. J. Neuroinflamm. 3:24. 10.1186/1742-2094-3-24 16959029 PMC1574293

[B42] Kim W. K. Ganea D. Jonakait G. M. (2002). Inhibition of microglial CD40 expression by pituitary adenylate cyclase-activating polypeptide is mediated by interleukin-10. J. Neuroimmunol. 126, 16–24. 10.1016/S0165-5728(02)00059-0 12020953

[B43] Kraft A. D. Harry G. J. (2011). Features of microglia and neuroinflammation relevant to environmental exposure and neurotoxicity. Int. J. Environ. Res. Public Health 8, 2980–3018. 10.3390/ijerph8072980 21845170 PMC3155341

[B44] Kumar A. Chen S. H. Kadiiska M. B. Hong J. S. Zielonka J. Kalyanaraman B.. (2014). Inducible nitric oxide synthase is key to peroxynitrite-mediated, LPS-induced protein radical formation in murine microglial BV2 cells. Free Radic. Biol. Med. 73, 51–59. 10.1016/j.freeradbiomed.2014.04.014 24746617 PMC4111989

[B45] Li J. O W. Li W. Jiang Z. G. Ghanbari H. A. (2013). Oxidative stress and neurodegenerative disorders. Int. J. Mol. Sci. 14, 24438–24475. 10.3390/ijms141224438 24351827 PMC3876121

[B46] Lin S. X. Lisi L. Dello Russo C. Polak P. E. Sharp A. Weinberg G. (2011). The anti-inflammatory effects of dimethyl fumarate in astrocytes involve glutathione and haem oxygenase-1. ASN Neuro 3, 75–84. 10.1042/AN20100033 21382015 PMC3072764

[B47] Lowry O. H. Rosebrough N. J. Farr A. L. Randall R. J. (1951). Protein measurement with the Folin phenol reagent. J. Biol. Chem. 193, 265–275. 14907713

[B48] Lu S. C. (2009). Regulation of glutathione synthesis. Mol. Aspects Med. 30, 42–59. 10.1016/j.mam.2008.05.005 18601945 PMC2704241

[B49] Luo X. G. Ding J. Q. Chen S. D. (2010). Microglia in the aging brain: relevance to neurodegeneration. Mol. Neurodegener. 5:12. 10.1186/1750-1326-5-12 20334662 PMC2852379

[B50] Mander P. K. Jekabsone A. Brown G. C. (2006). Microglia proliferation is regulated by hydrogen peroxide from NADPH oxidase. J. Immunol. 176, 1046–1052. 10.4049/jimmunol.176.2.1046 16393992

[B51] Manzardo A. M. He J. Poje A. Penick E. C. Campbell J. Butler M. G. (2013). Double-blind, randomized placebo-controlled clinical trial of benfotiamine for severe alcohol dependence. Drug Alcohol Depend. 133, 562–570. 10.1016/j.drugalcdep.2013.07.035 23992649 PMC3818307

[B52] Maral J. Puget K. Michelson A. M. (1977). Comparative study of superoxide dismutase, catalase and glutathione peroxidase levels in erythrocytes of different animals. Biochem. Biophys. Res. Commun. 77, 1525–1535. 10.1016/S0006-291X(77)80151-4 901548

[B53] Marnett L. J. Rowlinson S. W. Goodwin D. C. Kalgutkar A. S. Lanzo C. A. (1999). Arachidonic acid oxygenation by COX-1 and COX-2. Mechanisms of catalysis and inhibition. J. Biol. Chem. 274, 22903–22906. 10.1074/jbc.274.33.22903 10438452

[B54] Miljkovic D. BlaŽevski J. Petkovic F. Djedovic N. Momèilovic M. Stanisavljevic S.. (2015). A comparative analysis of multiple sclerosis-relevant anti-inflammatory properties of ethyl pyruvate and dimethyl fumarate. J. Immunol. 194, 2493–2503. 10.4049/jimmunol.1402302 25681336

[B55] Min K. J. Pyo H. K. Yang M. S. Ji K. A. Jou I. Joe E. H. (2004). Gangliosides activate microglia via protein kinase C and NADPH oxidase. Glia 48, 197–206. 10.1002/glia.20069 15390122

[B56] Morgan M. J. Liu Z. G. (2011). Crosstalk of reactive oxygen species and NF-κB signaling. Cell Res. 21, 103–115. 10.1038/cr.2010.178 21187859 PMC3193400

[B57] Moskaug J. Ø, Carlsen, H. Myhrstad M. C. Blomhoff R. (2005). Polyphenols and glutathione synthesis regulation. Am. J. Clin. Nutr. 81, 277S-283S. 15640491 10.1093/ajcn/81.1.277S

[B58] Navarro-Gonzálvez J. A. García-Benayas C. Arenas J. (1998). Semiautomated measurement of nitrate in biological fluids. Clin. Chem. 44, 679–681. 9510886

[B59] Pan X. Gong N. Zhao J. Yu Z. Gu F. Chen J.. (2010). Powerful beneficial effects of benfotiamine on cognitive impairment and beta-amyloid deposition in amyloid precursor protein/presenilin-1 transgenic mice. Brain 133, 1342–1351. 10.1093/brain/awq069 20385653

[B60] Park J. Choi H. Min J. S. Park S. J. Kim J. H. Park H. J.. (2013). Mitochondrial dynamics modulate the expression of pro-inflammatory mediators in microglial cells. J. Neurochem. 127, 221–232. 10.1111/jnc.12361 23815397

[B61] Qin H. Wilson C. A. Lee S. J. Zhao X. Benveniste E. N. (2005). LPS induces CD40 gene expression through the activation of NF-kappaB and STAT-1alpha in macrophages and microglia. Blood 106, 3114–3122. 10.1182/blood-2005-02-0759 16020513 PMC1895321

[B62] Rojo A. I. McBean G. Cindric M. Egea J. López M. G. Rada P.. (2014). Redox control of microglial function: molecular mechanisms and functional significance. Antioxid. Redox Signal. 21, 1766–1801. 10.1089/ars.2013.5745 24597893 PMC4186766

[B63] Roth T. L. Nayak D. Atanasijevic T. Koretsky A. P. Latour L. L. McGavern D. B. (2014). Transcranial amelioration of inflammation and cell death after brain injury. Nature 505, 223–228. 10.1038/nature12808 24317693 PMC3930079

[B64] Schmid U. Stopper H. Heidland A. Schupp N. (2008). Benfotiamine exhibits direct antioxidative capacity and prevents induction of DNA damage *in vitro*. Diabetes Metab. Res. Rev. 24, 371–377. 10.1002/dmrr.860 18384109

[B65] Schupp N. Dette E. M. Schmid U. Bahner U. Winkler M. Heidland A.. (2008). Benfotiamine reduces genomic damage in peripheral lymphocytes of hemodialysis patients. Naunyn Schmiedebergs Arch. Pharmacol. 378, 283–291. 10.1007/s00210-008-0310-y 18509620

[B66] Siomek A. (2012). NF-κB signaling pathway and free radical impact. Acta Biochim. Pol. 59, 323–331. 22855720

[B67] Solito E. Sastre M. (2012). Microglia function in Alzheimer's disease. Front. Pharmacol. 3:14. 10.3389/fphar.2012.00014 22363284 PMC3277080

[B68] Stirban A. Negrean M. Stratmann B. Gawlowski T. Horstmann T. Götting C.. (2006). Benfotiamine prevents macro- and microvascular endothelial dysfunction and oxidative stress following a meal rich in advanced glycation end products in individuals with type 2 diabetes. Diabetes Care 29, 2064–2071. 10.2337/dc06-0531 16936154

[B69] Sun M. Zigman S. (1978). An improved spectrophotometric assay for superoxide dismutase based on epinephrine autoxidation. Anal. Biochem. 90, 81–89. 10.1016/0003-2697(78)90010-6 727489

[B70] Talalay P. Dinkova-Kostova A. T. Holtzclaw W. D. (2003). Importance of phase 2 gene regulation in protection against electrophile and reactive oxygen toxicity and carcinogenesis. Adv. Enzyme Regul. 43, 121–134. 10.1016/S0065-2571(02)00038-9 12791387

[B71] Uttara B. Singh A. V. Zamboni P. Mahajan R. T. (2009). Oxidative stress and neurodegenerative diseases: a review of upstream and downstream antioxidant therapeutic options. Curr. Neuropharmacol. 7, 65–74. 10.2174/157015909787602823 19721819 PMC2724665

[B72] Valdovinos-Flores C. Gonsebatt M. E. (2012). The role of amino acid transporters in GSH synthesis in the blood-brain barrier and central nervous system. Neurochem. Int. 61, 405–414. 10.1016/j.neuint.2012.05.019 22634224

[B73] Van der Perren A. Macchi F. Toelen J. Carlon M. S. Maris M. de Loor H.. (2015). FK506 reduces neuroinflammation and dopaminergic neurodegeneration in an α-synuclein-based rat model for Parkinson's disease. Neurobiol. Aging 36, 1559–1568. 10.1016/j.neurobiolaging.2015.01.014 25660193

[B74] Verma S. Reddy K. Balakumar P. (2010). The defensive effect of benfotiamine in sodium arsenite-induced experimental vascular endothelial dysfunction. Biol. Trace Elem. Res. 137, 96–109. 10.1007/s12011-009-8567-7 19943121

[B75] Villacara A. Kumami K. Yamamoto T. Mrsulja B. B. Spatz M. (1989). Ischemic modification of cerebrocortical membranes: 5-hydroxytryptamine receptors, fluidity, and inducible *in vitro* lipid peroxidation. J. Neurochem. 53, 595–601. 10.1111/j.1471-4159.1989.tb07375.x 2746237

[B76] Wilms H. Sievers J. Rickert U. Rostami-Yazdi M. Mrowietz U. Lucius R. (2010). Dimethylfumarate inhibits microglial and astrocytic inflammation by suppressing the synthesis of nitric oxide, IL-1beta, TNF-alpha and IL-6 in an *in-vitro* model of brain inflammation. J. Neuroinflamm. 7:30. 10.1186/1742-2094-7-30 20482831 PMC2880998

[B77] Wu S. Ren J. (2006). Benfotiamine alleviates diabetes-induced cerebral oxidative damage independent of advanced glycation end-product, tissue factor and TNF-α. Neurosci. Lett. 394, 158–162. 10.1016/j.neulet.2005.10.022 16260089

[B78] Yang N. C. Ho W. M. Chen Y. H. Hu M. L. (2002). A convenient one-step extraction of cellular ATP using boiling water for the luciferin-luciferase assay of ATP. Anal. Biochem. 306, 323–327. 10.1006/abio.2002.5698 12123672

[B79] Zhang M. An C. Gao Y. Leak R. K. Chen J. Zhang F. (2013). Emerging roles of Nrf2 and phase II antioxidant enzymes in neuroprotection. Prog. Neurobiol. 100, 30–47. 10.1016/j.pneurobio.2012.09.003 23025925 PMC3623606

