# Supplementary figures and images for: Benfotiamine upregulates antioxidative system in activated BV-2 microglia cells
Source: Front Cell Neurosci. 2015 Sep 4;9:351. doi: 10.3389/fncel.2015.00351 (PMC4559599; doi:10.3389/fncel.2015.00351)

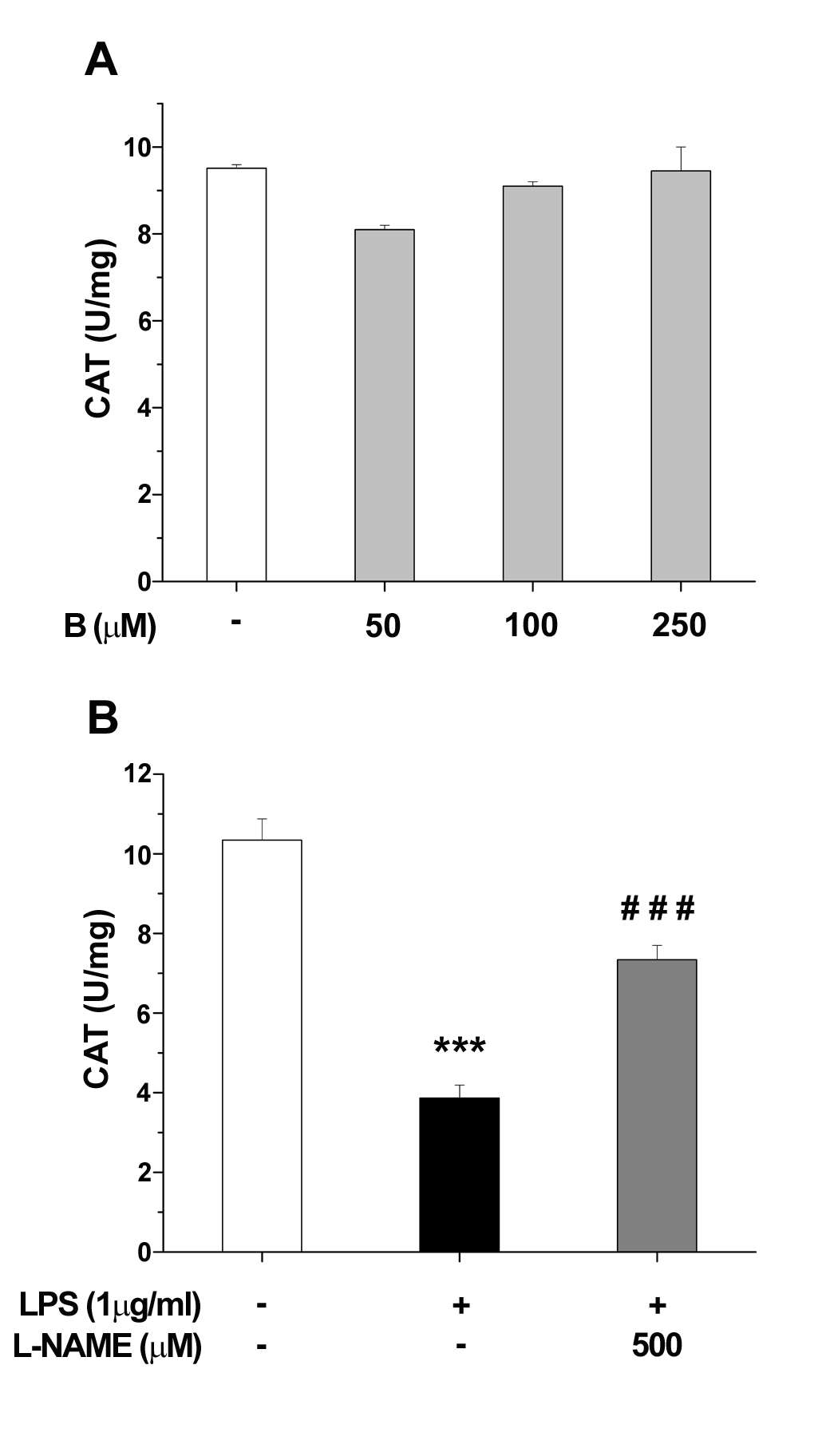

Supplement: Figure S1 — CAT activity—effect of benfotiamine in basal conditions and effect of iNOS inhibitor (L-NAME). Activity of CAT was determined 24 h after treatment with benfotiamine (50, 100 and 250 μM) in the absence of LPS stimulation (A). CAT activity was examined in BV-2 cells treated with L-NAME (500 μM) for 1 h and then stimulated with LPS for 24 h (B). The results are expressed as mean specific activities (U/mg) ± SEM from three independent cell preparations. ***p < 0.001 compared with control group, ###p < 0.001 compared with LPS treated group. [file Image1.TIF]

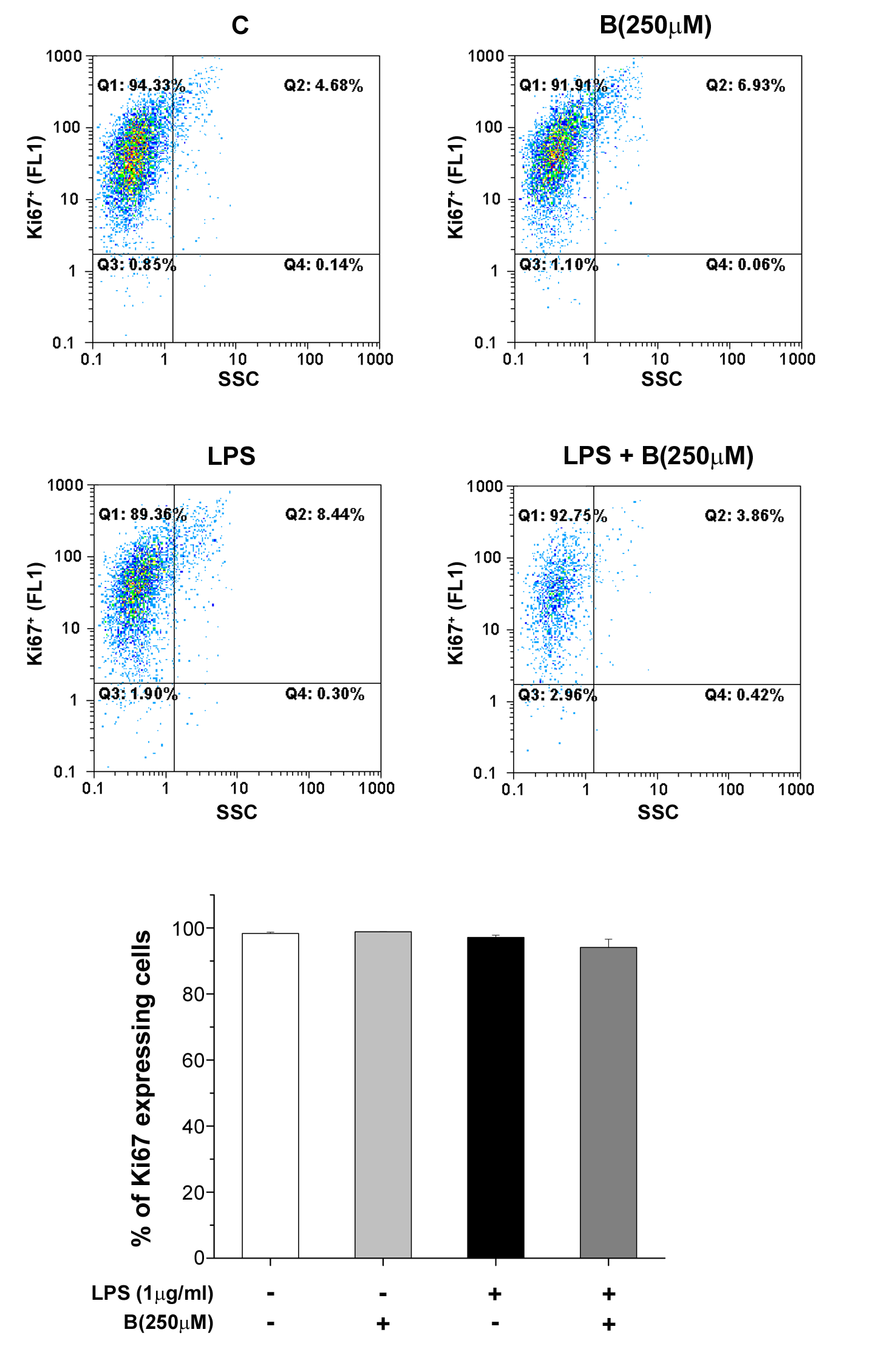

Supplement: Figure S2 — Effect of benfotiamine and LPS treatment on BV-2 cell proliferation. BV-2 cells were treated with benfotiamine (250 μM), LPS (1 μg/ml) or their combination for 24 h, stained with Ki-67 antibody and analyzed with FACS. Representative dot plots are shown. Statistical analysis was performed and mean values from three independent experiments are presented on the graph. [file Image2.TIF]
